# Genotype testing in HIV-infected pregnant women

**DOI:** 10.1186/1758-2652-13-S4-P145

**Published:** 2010-11-08

**Authors:** C Afonso, A Zagalo, N Janeiro, F Antunes

**Affiliations:** 1 Hospital de Santa Maria, Infectious Diseases, Lisbon, Portugal

## Background

Published guidelines recommend HIV genotype resistance testing for all pregnant women with detectable viraemia, regardless of antiretroviral therapy (ART) exposure, enabling to optimize ART.

## Objectives:

To identify different HIV subtypes in pregnant women and correlate them with epidemiological factors.

## Methods

HIV genotype resistance tests results were available on 50 HIV-infected pregnant women between 2003 and 2010.

## Results

The results showed a majority of cases of non-B subtypes: subtype G 42%, subtype B 24%, subtype C 14%, CRF02_AG 12%, subtypes A, D, CRFO3_BG and CRF01_AE 2%. Comparative study of the different subtypes showed epidemiological differences:

•Subtype G: 81,6% of women of Portuguese origin, 14,4% of African origin, with 76% of infection acquired by sexual transmission and 23,8% by intravenous drug use, 28,6% of women were on ART, and the median time of HIV infection was three and a half years.

•Subtype B: all of the women of Portuguese origin, with 83,3% of infection acquired by sexual transmission and 16,78% by intravenous drug use, 50% of women were on ART, and the median time of HIV infection was eight years.

•Subtype C: all of the women of African origin, all acquired the infection by sexual transmission, none of the women were on ART, and the median time of HIV infection was three years.

•CRF02_AG: 63,2% of women of Portuguese origin, 28,6% of African origin, with 57,1% of infection acquired by sexual transmission, 28,6% by intravenous drug use, and 14,3% by vertical transmission, 14,3% of women were on ART, and the median time of HIV infection was 2 years.

## Conclusions

The analysis of this data shows a bimodal distribution of the epidemic in Portugal: an initial epidemic with B strains, as in western Europe, and a second, latter one, with non-B subtypes, disseminated through patients from African origin and intravenous drug users. The presence on non-B strains, with intrinsic patterns of resistance, puts a burden on the management of these women.

